# Spontaneous Infiltration Behavior of Al Matrix into Carbon Fiber Bundles Induced by Al-Ni Binary Reaction

**DOI:** 10.3390/ma18050966

**Published:** 2025-02-21

**Authors:** Jiaming Liu, Xi Yang, Shichao Liu, Junjia Zhang, Yubo Zhang, Tingju Li

**Affiliations:** 1 State Key Laboratory of Advanced Equipment and Technology for Metal Forming, Shandong University, Jinan 250061, China; liu.jm@sdu.edu.cn; 2 Key Laboratory for Liquid-Solid Structural Evolution and Processing of Materials (Ministry of Education), Shandong University, Jinan 250061, China; 3 Key Laboratory of Solidification Control and Digital Preparation Technology (Liaoning Province), Dalian University of Technology, Dalian 116024, China; yangxi@mail.dlut.edu.cn (X.Y.); tjuli@dlut.edu.cn (T.L.); 4 School of Iron and Steel, Soochow University, Suzhou 215006, China; 5 Key Laboratory of Lightweight Structural Materials (Liaoning Province), Northeastern University, Shenyang 110819, China; zhangjj@mail.neu.edu.cn

**Keywords:** CF/Al composite, surface metallization, spontaneous infiltration behavior, synchrotron X-ray radiography, interfacial microstructure evolution

## Abstract

In this study, a Ni-coated carbon fiber reinforced Al-matrix (Ni-CF/Al) composite is prepared utilizing a pressure-free infiltration process. The CFs are coated with a layer of Ni through an electroless plating process, which facilitates the spontaneous infiltration behavior driven by the Al-Ni binary reaction. The spontaneous infiltration process, observed via synchrotron radiation in the direction opposite to gravity, demonstrates a fastest velocity of 31.02 ± 1.08 μm/s. By increasing the infiltration temperature, the interfacial microstructure of the composite can be enhanced, characterized by a reduction in un-infiltrated defects and promoted by the interfacial Al-Ni reaction. Notably, large-size Al-Ni intermetallic compounds (IMCs) at the interface are replaced by fine (Al+Al_3_Ni) eutectic structure, given an optimal fabrication temperature of 720 °C. This contributes to a significantly enhanced ultimate tensile strength (UTS) of the composite, reaching a maximum of 135 ± 4 MPa, which is 159.6% higher than that of the matrix.

## 1. Introduction

Carbon fiber reinforced Al-matrix (CF/Al) composites have attracted wide attention in transportation and aviation industries due to their excellent properties, including low density, high specific strength, and low thermal expansion coefficient. However, the fabrication of these composite remains challenging [1,2,3,4]. For instance, the infiltration of Al into CF bundles cannot spontaneously occur due to the poor interfacial wettability [5].

Applying metal coatings, such as Cu and Ni, to the surface of the CFs is an effective strategy to enhance wettability [6,7]. Previous studies have primarily explored the CF/Al interfacial wettability through titration tests using Al melt and graphite [8]. However, these methods are limited to observing changes in contact angle, and in situ infiltration observation has been rarely studied. In our previous work, the infiltration of Al melt into Ni-coated CFs was observed by synchrotron X-ray radiography [9]. Despite this progress, the mechanism governing spontaneous infiltration under gravitational effects is still unclear.

The optimization of fabrication methods has also been developed to address the aforementioned challenges. It had been demonstrated that at temperatures exceeding 1000 °C, the wettability between Al melt and CFs was improved, with contact angles decreasing to below 90° [10,11]. On this basis, a high-temperature fabrication process has been employed, while the harmful Al_4_C_3_ phase would be formed at the Al/C interface under these conditions [11,12]. High-pressure conditions were also applied to fabricate CF/Al composites at relatively low temperatures [13,14]. However, the applied external pressure might make it challenging to regulate the interfacial strength. Moreover, typical brittle fracture behavior of the composite would occur due to excessive interfacial strength, adversely affecting the composite performance [15]. Therefore, it is critical to develop a fabrication method under relatively low temperatures and pressures.

In this study, the infiltration process of Al melt into Ni-coated CFs in the anti-gravity direction was innovatively explored using synchrotron X-ray radiography. The spontaneous infiltration behavior, driven by the Al-Ni interfacial reaction, was revealed visually. On this basis, CF/Al composites were successfully fabricated through a pressure-free infiltration process at various temperatures.

## 2. Experimental

**Figure 1:**
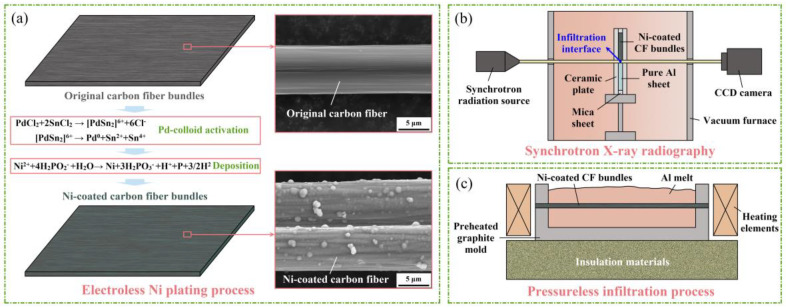
Schematic diagram of the (**a**) electroless Ni plating, (**b**) synchrotron radiation, and (**c**) fabrication process of the CF/Al composite.

Pure Al and unidirectional CF bundles (T300-3k, Toray Industries Inc., Tokyo, Japan) were utilized as the matrix and reinforcement, respectively. An electroless plating method was used to coat Ni onto the CF surfaces (Figure 1a). The specific details of the deposition process during electroless plating are selected based on our previous study [9]. To observe the infiltration process of molten Al in the anti-gravity direction in vacuum, in situ synchrotron radiation was performed using an X-ray beam with 25 keV energy and a charge-coupled device (CCD) camera (Figure 1b). The Ni-coated CF bundles were arranged atop the Al sheet and tightly connected with it. The infiltration couple was heated to 750 °C at a rate of 10.0 ± 0.2 °C/min and held for 10 min to ensure sufficient infiltration, according to our previous study [16]. Based on the synchrotron radiation experiments, Ni-CF/Al composites were prepared using a pressure-free infiltration method (Figure 1c). Molten Al was poured into a graphite mold containing the fixed CF bundles, followed by infiltration processes in a heat-treatment furnace at various temperatures determined through several experimental attempts (690 °C, 720 °C, and 750 °C) for 6 min that were deemed to be optimal [16]. Additionally, CF/Al composites were prepared in parallel at 750 °C.

The surface morphology of the CFs was observed using an IT800-SHL scanning electron microscope (SEM) (Tokyo, Japan). The microstructure investigation of the composites was performed by SEM, JXA-8530 electron probe microanalysis (EPMA) (Tokyo, Japan), and FEI Talos·F200X transmission electron microscopy (TEM) (Waltham, MA, USA). The density of the composites was measured based on Archimedes principle, with each sample configuration measured five times to ensure accuracy. The sizes of the IMCs were statistically analyzed, with 20 measurements taken for each sample to ensure data reliability. The tensile strength of the composites with a diameter of 5.0 ± 0.1 mm and a gauge length of 15.0 ± 0.1 mm was studied using a UTM 5105 universal testing machine (Kyoto, Japan) at a rate of 0.50 ± 0.02 mm/min. Each configuration of the composite sample was tested 3 times. The volume fraction of the CFs in the composite was approximately 9.0 ± 0.3%.

## 3. Results and Discussion

**Figure 2:**
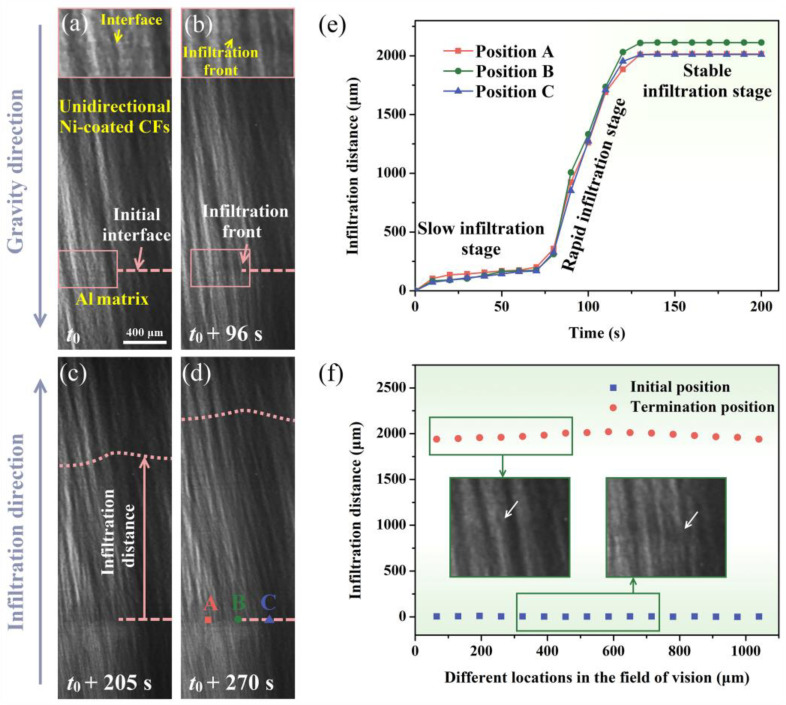
Time-evolved infiltration images for the Ni-coated CFs/Al couple in the direction opposite to gravity: (**a**) *t*_0_; (**b**) *t*_0_ + 96 s; (**c**) *t*_0_ + 205 s; (**d**) *t*_0_ + 270 s; (**e**) infiltration distance versus time curves at various positions; (**f**) initial and termination positions of the infiltration front.

Figure 2 presents the synchrotron radiation images of the Ni-coated CF/Al infiltration couple. The initial interface was relatively straight and clearly visible at *t*_0_ (627.0 ± 0.2 °C), indicating that no infiltration for the Al melt had yet occurred (see Figure 2a). At *t*_0_ + 96 s (643.0 ± 0.2 °C), the clearly visible interface disappeared, while a fuzzy region of Al could be observed and began to move upward, which was deemed as the infiltration front (Figure 2b). At this moment, the achieved temperature exceeding the Al-Ni eutectic temperature resulted in the occurrence of the eutectic reaction, even though the Al had not fully melted. At *t*_0_ + 205 s (661.0 ± 0.2 °C), the infiltration front adopted an undulating shape and had moved significantly upward, away from the initial position in the direction opposite to gravity, demonstrating a spontaneous infiltration process due to significantly promoted CF/Al interfacial wettability (Figure 2c). This phenomenon can be attributed to the positive capillary pressure (*P*_c_) caused by the low contact angle between the Al melt and Ni coatings, which was lower than 90° [8], as described by Equations (1) and (2) [17,18,19]:

(1) $$P_{c}=\frac{2\gamma \cos\theta }{d}$$

(2) $$h={\left[\frac{2F(Pc-Pi)}{\eta }\right]}^{1/2}t^{1/2}$$

where *γ*, *θ*, and *d* are the surface tension, contact angle, and the equivalent capillary radius, respectively, and *h* is the infiltration distance. *P*_i_ is the passage resistance. *F* and *η* are the osmotic coefficient and coefficient of viscosity, respectively.

As the infiltration process completed at *t*_0_ + 270 s (672.0 ± 0.2 °C), the infiltration fronts exhibited minimal changes (Figure 2d). Three randomly selected points were used to calculate the infiltration velocity and distance, see Figure 2d. The infiltration distance versus time is shown in Figure 2e, with *t*_0_ + 96 s defined as the starting point (0 s). Based on the variation in infiltration velocity, the infiltration process can be then divided into three distinct stages: slow, rapid, and stable infiltration. In the initial slow infiltration stage, the relatively poor interfacial reaction below the melting point temperature resulted in a low infiltration velocity of 1.59 ± 0.06 μm/s. Subsequently, the rapid infiltration stage was observed between 70.0 ± 0.4 s and 130 ± 0.5 s (*t*_0_ + 166.0 ± 0.4 s~*t*_0_ + 226.0 ± 0.5 s), during which the average infiltration velocity significantly increased to approximately 31.02 ± 1.08 μm/s. In the final stable infiltration stage, the velocity gradually decreased to nearly 0 μm/s. Simultaneously, the infiltration distances of the undulating front were quantitatively calculated (Figure 2e), revealing a maximum infiltration distance of approximately 2020 ± 10 μm, with an average value of 1978 ± 27 μm. These results demonstrate that the application of Ni coatings enables the spontaneous infiltration of molten Al into CF bundles, driven by the Al-Ni binary reaction.

**Figure 3:**
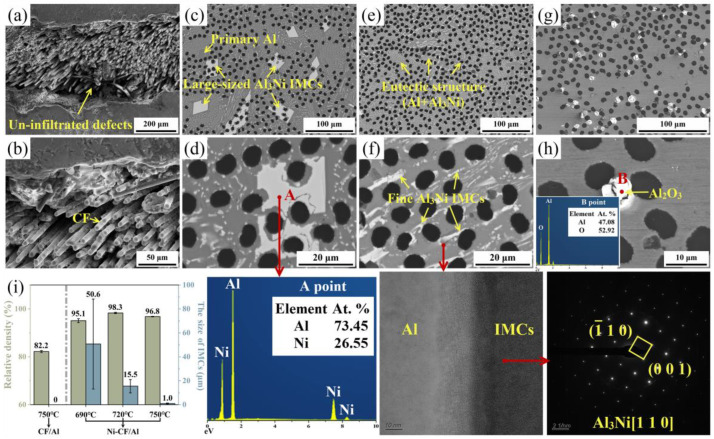
Micromorphologies of (**a**,**b**) CF/Al composites prepared at 750 °C and Ni-CF/Al composites prepared at (**c**,**d**) 690 °C, (**e**,**f**) 720 °C, and (**g**,**h**) 750 °C; (**i**) the relative density of the composites fabricated at varying temperatures and the size of IMCs.

Figure 3a displays the microstructure of CF/Al composites without Ni coatings on the CFs. Due to the poor interfacial wettability, molten Al cannot spontaneously infiltrate into the CF bundles, resulting in the generation of considerable un-infiltrated defects within the CF bundles. Figure 3c–h shows the microstructure of Ni-CF/Al composites fabricated at various infiltration temperatures. At 690 °C, the infiltration of Al melt was significantly promoted, resulting in much fewer defects within the CF bundles, as shown in Figure 3c,d. Simultaneously, larger-size Al-Ni IMCs formed primarily near CFs due to interfacial Al-Ni reactions. The element composition of these IMCs was 74.6 ± 1.2 at.% Al and 25.4 ± 1.2 at.% Ni (typical IMC is shown at Point A), which was determined to be Al_3_Ni. Additionally, fine Al_3_Ni with eutectic structure was also found [20]. As the temperature increased to 720 °C, the large-sized Al_3_Ni phases disappeared and totally transformed to fine eutectic (Al+Al_3_Ni) phases. This transformation was attributed to further enhanced Al-Ni interfacial diffusion (Figure 3e,f). The Al_3_Ni phase was further confirmed by HR-TEM images at the IMC/Al interface and corresponding electron diffraction patterns (Figure 3e). As temperature increased to 750 °C, the fine Al_3_Ni phases were gradually reduced owing to the rapid diffusion of Ni (Figure 3g,h). Notably, apparent white impurities could be found within the CF bundles, likely owing to the difficulty for impurities to be removed from CF bundles. The composition of the white impurities was 42.6 ± 2.3 at.% Al and 57.4 ± 2.3 at.% O according to the EDS analysis results (typical impurity is shown at Point B), identifying the impurity as Al_2_O_3_.

Subsequently, the relative density of the composites fabricated at varying temperatures and the size of the IMCs were measured and are summarized in Figure 3i. Among them, the green columns and labels in Figure 3i represent the relative density of the composites, while the blue columns and labels correspond to the size of the IMCs. By the introduce of Ni coatings onto the CF surfaces, the relative density of the composites significantly increased to 95.1 ± 0.8%, 98.3 ± 0.3%, and 96.8 ± 0.1% at 690 °C, 720 °C, and 750 °C, respectively, compared to that of the composites with desized CFs (82.2 ± 0.4%). Furthermore, the relatively inhomogeneous IMCs with a larger size of 50.6 ± 37.6 μm was noted for the Ni-CF/Al composites fabricated at 690 °C, while gradually decreased size of the IMCs was obtained as the fabrication temperature increased to 720 °C (15.5 ± 5.6 μm) and 750 °C (1.0 ± 0.4 μm). These findings were consistent with the microstructure evolution results described earlier.

**Figure 4:**
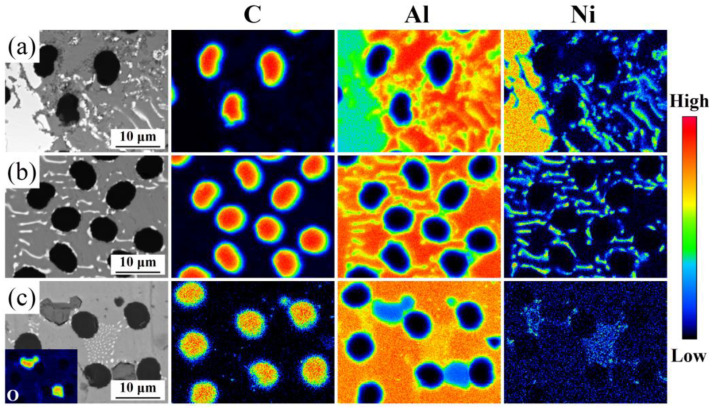
Element distribution in the composites prepared at (**a**) 690 °C, (**b**) 720 °C, and (**c**) 750 °C.

The element distribution within the CF/Al composite was further investigated, as shown in Figure 4. With the increased infiltration temperature from 690 °C to 750 °C, the Al-Ni diffusion could be continuously promoted during the pressure-free infiltration process of the composite. Specifically, the uniformity of Ni element distribution was significantly improved with the increasing infiltration temperature. Accordingly, this resulted in a transformation from large-sized Al-Ni IMCs to a fine eutectic structure (Al+Al_3_Ni), aligning well with the aforementioned microstructure analysis results at the CF/Al interface regions. The interfacial microstructure evolution can be attributed to the diffusion behavior of Ni into Al melt. It had been demonstrated that the Al-Ni interfacial diffusion could be promoted with the increase in fabrication temperature [21]. In such a case, the diffusion distance of Ni into Al melt would be improved, leading to a relatively lower localized Ni content. This phenomenon, as per the Al-Ni binary phase diagram [22], accounts for the morphological evolution of Al-Ni IMCs and the improved homogeneity in their distribution. In particular, the large-size primary Al_3_Ni at the interface changed to a fine (Al+Al_3_Ni) eutectic structure.

**Figure 5:**
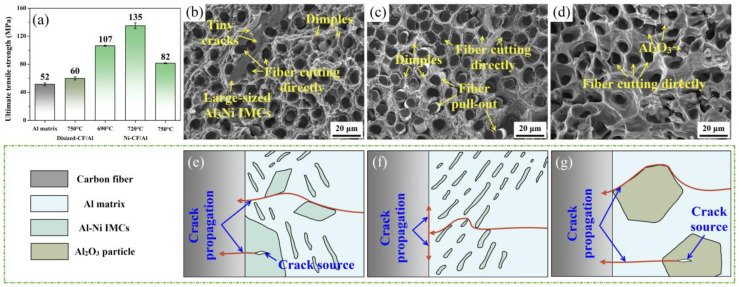
(**a**) Average UTS of CF/Al composites; fracture morphologies of Ni-CF/Al composites prepared at (**b**) 690 °C, (**c**) 720 °C, and (**d**) 750 °C; (**e**–**g**) corresponding schematic diagram of the crack propagation.

Figure 5a shows the UTS of the composites. The UTS of the CF/Al composites was measured at 60 ± 2 MPa. It increased to 107 ± 1 MPa, 135 ± 4 MPa, and 82 ± 1 MPa for the Ni-CF/Al composites with the increase in infiltration temperature, respectively, which was 105.8%, 159.6%, and 57.7% higher than the Al matrix (52 ± 2 MPa). In this case, the infiltration temperature of 720 °C was identified as optimal. The fracture morphology of composites and a schematic diagram of crack propagation at the interface are shown in Figure 5b–g. Notably, the interfacial failure behavior would significantly affect the composite strength [23]. For composites fabricated at 690 °C, the brittle fracture of large-sized Al_3_Ni IMCs was observed, accompanied by the fibers cutting directly (Figure 5b). This phenomenon is attributed to the formation of large-sized Al_3_Ni IMCs, which are prone to initiating crack sources and leading to rapid failure of fibers (Figure 5e) [24]. This resulted in a larger multiple fracture number (*k*) of fibers, which was proved to lower the harmful energy absorption and negatively affect the strength of the composite, according to Equation (3) [25].

(3) $$\sigma_{c}=\min[{\left(\frac{L_{0}}{2(\ln2)e\beta \lambda k}\right)}^{\frac{1}{\beta }}\gamma ,{\left(\frac{2L_{0}\tau_{b}}{e(\beta +1)R_{f}k}\right)}^{\frac{1}{\beta +1}}\gamma^{\frac{\beta }{\beta +1}}]$$

where *σ*_c_ is the strength of the composite. *L*_0_ is the reference length, *e* is the natural constant, and *γ* and *β* are the scale and shape parameters, respectively. *λ* is the ineffective length factor, and *k* is the multiple fracture number. *τ_b_* is the strength of the interface, and *R*_f_ is the fiber radius.

For the case of 720 °C, the fracture morphology exhibited phenomena such as fiber pull-out and fiber cutting directly, accompanied by the presence of more dimples, as shown in Figure 5c. In this scenario, tiny cracks can be deflected by the eutectic structure (Al+Al_3_Ni), leading to a continuously changed crack propagation path and more energy absorption (see Figure 5f) [26]. Consequently, effective load transferred to the CFs can be obtained, contributing the highest composite strength. As the temperature further increased to 750 °C, the primary failure mechanisms shifted to fiber cutting directly and broken Al_2_O_3_ (Figure 5d). Notably, the Al_2_O_3_ impurities acted as crack sources, resulting in rapid crack propagation across the fibers and relatively poor load transfer (Figure 5g). In this case, the strength of the composite instead decreased.

Table 1 presents a comparison of preparation temperature, pressure, and the improvement in UTS relative to the matrix for the composite in this work and those reported in the literature. It can be found that achieving a significant improvement in UTS compared to the matrix typically requires the application of relatively high pressure. In contrast, this study demonstrates a significant improvement in the UTS of the CF/Al composite under pressure-free conditions, even with a much lower CF volume fraction. This eliminates the need for additional pressure during composite preparation, thereby reducing the cost of the preparation equipment. Moreover, the relative simplicity of the process allows for improved efficiency of the composite preparation. The aforementioned factors lead to a potential decrease in the cost of obtained composite parts. Additionally, it was also noticed that for most studies reported in the literature, there were no apparent interfacial IMCs generated at the CF/matrix interface. Despite the localized Al-rich IMCs identified in some research, they have not been further identified or regulated. In the present study, effective Al-Ni IMC regulation and morphology optimization were achieved, facilitating a considerable improvement in the mechanical properties of the CF/Al composite.

## 4. Conclusions

In this study, the infiltration behavior of molten Al into Ni-coated CF bundles was in situ investigated in the direction opposite to gravity. Spontaneous infiltration behavior of the Al melt could be obtained, driven by the Al-Ni binary reaction. The largest infiltration velocity was measured at 31.02 ± 1.08 μm/s, with an average infiltration distance of 1978 ± 27 μm. On this basis, the Ni-CF/Al composite was successfully fabricated using a pressure-free infiltration process. With the increase in infiltration temperature, Ni diffusion could be improved, leading to the elimination of defects and a transformation in the morphology of Al-Ni IMCs from large-size primary Al_3_Ni to fine (Al+Al_3_Ni) eutectic structure. This resulted in an effective load transfer and crack propagation deflection, contributing to a significantly enhanced UTS of the Ni-CF/Al composite (135 ± 4 MPa) at the optimal infiltration temperature of 720 °C, representing a 159.6% increase compared to the Al matrix. These findings proved a promising pressure-free fabrication route for fiber/metal composites with enhanced interfacial and mechanical performance.

## Figures and Tables

**Table 1:** Comparison of preparation temperature, pressure, and improvement in UTS compared to the matrix for the composite in this work and those reported in the literature.

Ref.	CF	CF Volume Fraction/%	Matrix	Preparation Method	Temperature/°C	Pressure/MPa	Interfacial IMCs	Improvement in UTS/MPa	Percentage of UTS Improvement/%
[14]	NA	20	2024Al	Squeeze casting	750	50	Al-Ni IMCs	168	66.4
[27]	T700	45	AZ91D	Squeeze casting	610	30	/	103	68.7
[28]	T300	NA	A-5Cu-0.5Mn	Plasma sintering	480	50	/	34	20.2
[29]	NA	2	Al-17Si	Liquid metallurgy	710	/	/	14	9.7
This study	T300	9	Al	Pressure-free infiltration	720	/	Al_3_Ni	83	159.6

## Data Availability

The original contributions presented in this study are included in the article. Further inquiries can be directed to the corresponding authors.
